# Advantages and disadvantages of using more sustainable ingredients in fish feed

**DOI:** 10.1016/j.heliyon.2022.e10527

**Published:** 2022-09-06

**Authors:** Beate Zlaugotne, Jelena Pubule, Dagnija Blumberga

**Affiliations:** Institute of Energy Systems and Environment, Riga Technical University, Azenes Street 12/1, Riga, Latvia

**Keywords:** Sustainability, Fish feed, Alternatives to ingredients, Salmon

## Abstract

As the population grows, demand for food increases. Fish is considered to be one of the most efficient sources of protein. But as demand increases, we need to think about the efficient and sustainable fish feed. There is a need to replace existing feed ingredients such as fishmeal and fish oil with more sustainable sources of protein and oil. In 1990, fish feed consisted mainly of fishmeal and fish oil, but today’s fish feed is dominated by vegetable protein and vegetable oil. Comparing the advantages and disadvantages of the alternatives is concluded that previously used fish feed ingredients such as fishmeal and fish oil are not the most efficient, sustainable, and economically viable resources. The comparison shows why the composition of fish feed has shifted from 1990 to 2020 towards the use of plant resources in fish feed, as plant resources are more efficient, sustainable, and economically viable.

## 1 Introduction

Sustainability is essential in every sector to avoid resource depletion, achieve an ecological balance, and use resources efficiently. Society’s interaction with the environment is deteriorating at an accelerating rate, where various global environmental, social, and economic problems are emerging [1]. Ensuring sustainability is a challenge to maintaining the ecological balance, where economic growth and environmental quality improvements are needed. People influence sustainability through their choices, as they contribute by choosing a more sustainable product or service.

As the population grows, from 7,16 billion in 2012 to 7,91 billion in 2021, an efficient food system that can feed the population in an environmentally and sustainably sustainable way is necessary [2]. In the food sector, people have access to a wide range of products and can choose more sustainable products, influence their food consumption and dispose of them efficiently instead of throwing them away. A valuable nutrition source with a relatively low environmental impact is blue food – aquatic animals, plants, or algae [3]. In 2019, the average person ate 710 kg of food, most of it was vegetables and fruit, but animal protein such as seafood, poultry, pork, and beef accounted for 9% of the total diet [4].

Fisheries and aquaculture production reaches an all-time high of 214 million tons in 2020 [5]. We are eating more aquatic food than ever before - around 20.2 kg per capita in 2020 and the consumed level is double than it was 50 years ago [5]. Forecast in the fish production sector shows that fish production will grow at an annual rate of 1.2% by 2030, with 90% of fish production going to food and 10% used to mainly produce fishmeal and fish oil by 2030 [6].

One of the main species produced in world aquaculture is Atlantic salmon, from 2380.2 thousand tons of live weight in 2015–2719.62 thousand tons of live weight in 2020 [5]. About 80% of the world’s salmon harvest is farmed in large nets in protected waters such as fjords or bays, and the majority of farmed salmon comes from Norway, Chile, Scotland, and Canada [7]. Salmon farming is the most advanced form of large-scale intensive aquaculture and is effectively used to transform marine resources into high-quality food available all year round [8]. Farmed Atlantic salmon is a versatile and popular product that meets the needs of today's consumers [9]. Also, farmed seafood is an efficient source of protein [10].

Due to the increasing demand for animal protein, this will also lead to an increase in feed ingredients such as fishmeal and fish oil, which are available in limited quantities, and it is essential to develop feed that is sustainable and based on non-food resources [11]. The study aims to make a qualitative comparison between fishmeal and protein source alternatives and to compare fish oil with other oil alternatives used in fish feed production.

## 2 Methods

**Figure 1:**
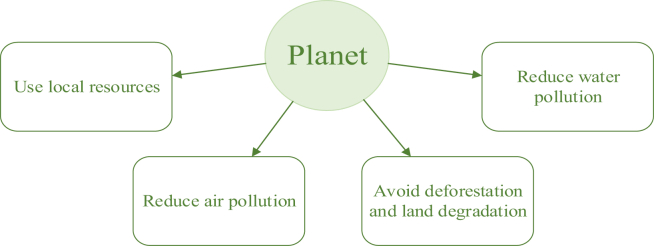
Elements of sustainable animal feed production were prioritised according to the sustainability dimension – planet [12].

The Food and Agriculture Organization (FAO) has made a working paper on “Identification of indicators for evaluating the sustainability of animal diets” based on a survey where participated academics, industry, farmers' associations, government organizations, and non-governmental organizations (NGOs), and inter-governmental organizations [12]. Sustainable animal diets are based on the planet, people, and profit dimension and aspects such as resource efficiency, environmental protection, and social and economic benefits [12]. For the planet dimension, the most popular indicators are in Figure 1. Also, popular indicators are improving the sector's resilience to natural disasters, improving or at least not reducing biodiversity, and leaving a minimal carbon footprint [12].

**Figure 2:**
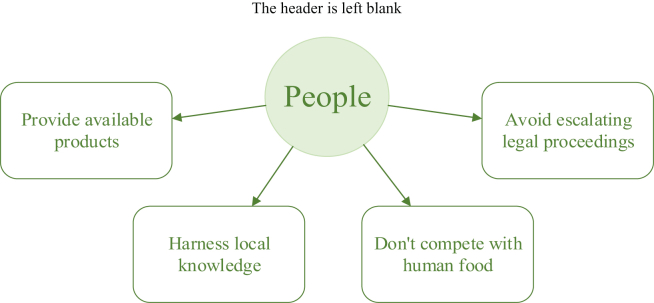
Elements of sustainable animal feed production were prioritised according to the sustainability dimension – people [12].

Figure 2 shows the most popular indicators for the people dimension. Other indicators such as the social aspects of farming, not being culturally offensive to producers and consumers of animal products, and being part of corporate social policies were also quite popular in the survey [12].

**Figure 3:**
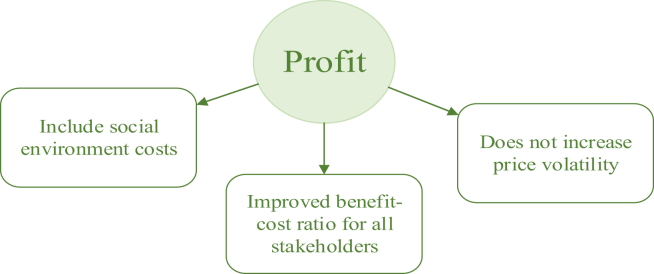
Elements of sustainable animal feed production were prioritised according to the sustainability dimension – profit [12].

Survey results on the profit dimension are in Figure 3 were, where the most famous indicator is the environmental and social costs of negative externalities such as environmental degradation, greenhouse gas emissions, and biodiversity loss [12].

Two critical components of sustainable fish feed are feed efficiency and feed ingredients, as feed can provide the best health and performance for fish [7]. Feed efficiency is an important indicator, as high-quality feed and effective fisheries management can reduce the amount of feed used and result in better fish growth from less feed consumed [13]. Feed ingredients have an impact on fish and the environment, as it is necessary to evaluate feed materials and whether or not an alternative is possible that would be more effective and have less impact on the environment.

Historically, fishmeal and fish oil were considered the two most essential ingredients in salmon feed due to their valuable nutrient composition [8]. Because the growth of aquaculture has led to a dependence on limited feed ingredients, particularly fishmeal and fish oil, alternatives are being explored and used as technically and economically feasible [8].

**Figure 4:**
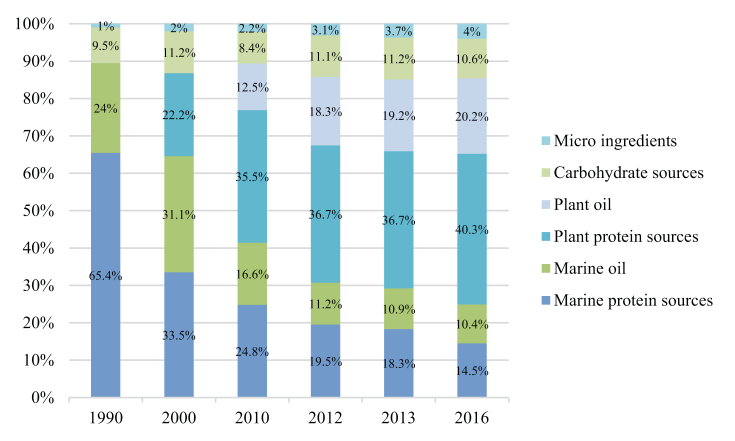
Ingredients (% of feed) in Norwegian salmon feed [14].

**Figure 5:**
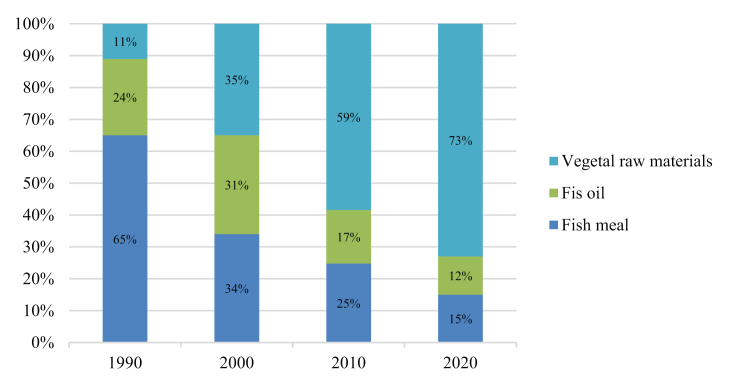
Ingredients (% of feed) in Norwegian salmon feed from company Mowi [7].

Figure 4 and 5 summarize the percentage changes in dietary ingredients for Norwegian salmon from 1990. Salmon diets have changed over the years, and marine ingredients are being replaced by alternatives to plant ingredients.

The use of marine proteins is declining and accounted for only 14.5% of feed in 2016 (Figure 4). However, plant protein increased to 40.3% in 2016, and 1990 salmon feed was not a feed ingredient at all. Carbohydrates are added as a binding agent in salmon feed and have been present in pretty similar proportions over the period 1990–2016, making up on average 10.3% of the feed. The micro-ingredients in the feed are slowly increasing and include mixtures of vitamins and minerals, phosphorus sources, astaxanthin, and crystalline amino acids [14].

According to Mowi (producer of Atlantic salmon), the composition of salmon feed has also changed. The 1990 feed was dominated by fishmeal (65%), and in the 2020 feed is dominated by vegetable raw materials (73%).

## 3 Results and discussion

Fish is a good source of protein and omega-three fatty acids, which also benefit human health, so fish food must be of good quality for fish and humans afterward (Table 1.). Protein is more important for fish in the early stages of growth and as they are growing, protein requirements decreases [15].

**Table 1:** Benefits for fish and humans [16, 17, 18, 19]

	Impact to fish	Impact to human
Vitamin A	‒ essential micronutrient ‒ an important role as an immunostimulant	‒ immune function ‒ growth ‒ vision
Vitamin D	‒ skeletogenesis ‒ ossification	‒ to prevent bone diseases ‒ immune function
Vitamin B12		‒ impact for new cell development
Iron	‒ for biological reactions	‒ production of hemoglobin
Zinc	‒ for metalloenzymes	‒ immune function ‒ growth
Calcium	‒ for bone health	‒ for bone health ‒ in pregnancy
Selenium	‒ an essential trace element	‒ an essential trace element ‒ plays a role of an antioxidant ‒ stimulates the immune system
Omega-3		‒ slows down the development of cardiovascular diseases
Iodine	‒ for thyroid hormones	‒ to maintain normal metabolism
Protein	‒ for energy	‒ essential amino acid ‒ necessary for cell
Potassium	‒ for acid–base balance ‒ for osmoregulation	‒ for the nervous system ‒ muscle function ‒ heart rate

Feed ingredients not only have an impact on fish, but also on the environment, as it is necessary to evaluate feed materials, as well as whether or not an alternative is possible that would be more effective and have less impact on the environment. The fish feed alternative must be high quality and high nutritional value (omega-3 fatty acids, high protein content, adequate amino acids, digestibility, and taste), as well as insoluble carbohydrates, fiber and heavy metals need to be low because it affects the fish growth process and affects low feed conversion ratio, feed costs must be economically justified and feed production [20].

The use of insects in fish feed production is considered to be one of the most sustainable and economically viable alternatives [21]. Also, insect meal is rich in polyunsaturated fatty acid (PUFA) which is one of the healthy fats [22].

**Black Soldier Fly** is considered to be a more suitable fish meal alternative than plant-based alternatives, especially when considering specifically the gut health of fish [22].

The **mealworm** industry is evolving from a manual sector that is not very efficient to one that is becoming more efficient and profitable [23].

**Soybean** flour contains crude protein that makes the feed easily digestible, more sustainable than fishmeal, balanced amino acid content, and low price, but there is a possibility that biologically active compounds can affect fish health, growth, and reproductive development [24].

**Fishmeal** and fish oil are derived from wild fish, but catches are limited in a number of inseparable ways, so more sustainable solutions and alternatives are being sought [20].

To better compare the alternatives are summarized advantages and disadvantages of fish feed alternatives for protein sources in Table 2.

**Table 2:** Advantages and disadvantages of fish feed alternatives – protein source.

	Advantages	Disadvantages	Ref.
Black Soldier Fly	- food waste (vegetable, fruit, factory waste and animal tissues) can be converted into high quality protein - contains a high amount of protein - good lipid source - a well-balanced amount of amino acids - good source of minerals and vitamins (iron, zinc, potassium, phosphorus, manganese, magnesium) - palatability - sustainability - nutraceutical benefits - a valuable source of protein and amino acid - grow and multiplies rapidly - no arable land is required - effectively converts low-quality organic matter into high-quality proteins and fats	- price - an unbalanced diet, too much of an insect meal can negatively affect growth - the nutritional value of the feed and the effect on the fish vary depending on the species of insect	[21, 25, 26, 27]
Meal worm	- palatability - sustainability - nutraceutical benefits - a valuable source of protein and amino acid - grow and multiplies rapidly - no arable land is required - effectively converts low-quality organic matter into high-quality proteins and fats	- price - an unbalanced diet, too much of an insect meal can negatively affect growth - the nutritional value of the feed and the effect on the fish vary depending on the species of insect	[28, 29, 30]
Soybean	- high protein content - improves fish growth - price - availability	- lectin and non-starch polysaccharides reduced feed intake - low phosphorus content - the presence of indigestible fibers - lack of essential amino acids that affect the quality of fish - low in methionine - poor palatability - no longer sustainable - mycotoxin risk	[15, 20, 25, 27]
Fishmeal	- improves the growth of fish - pleasant taste - easily digests - balanced nutrition - composition and concentration of proteins, minerals, essential fatty acids and essential amino acids - low feed conversion factor, resulting in less feed waste - increased immunity, which improves survival - palatability	- no longer sustainable - availability - price	[20, 25]

In Table 3 are comparison of alternatives according to their mineral values and in Table 4 are advantages and disadvantages.

**Table 3:** Comparison of protein sources according to their mineral values [31, 32, 33, 34, 35, 36, 37, 38, 39, 40, 41, 42, 43, 44, 45]

	Black Soldier Fly	Meal worm	Soybean	Fishmeal
Iron (mg/kg)	100–630	9.61–245	92,9–919	81–715
Zinc (mg/kg)	42–300	33.8–117.4	41,4–77,0	56–381
Magnesium (mg/kg)	2100–5610	620–2027	2550–4940	700–4000
Calcium (mg/kg)	5360–61,620	156–435	1600–4660	11,800–80,100
Phosphorus (mg/kg)	6800–13,220	2640–7061	5640–7660	1530–43,400
Sodium (mg/kg)	890–2500	225–3644	60–1090	3200–19,800
Potassium (mg/kg)	10,200–18,790	3350–9480	20,200–25,200	330–15,700
Copper (mg/kg)	7.5–34.25	8.3–20	9,0–18,7	3–108
Manganese (mg/kg)	190–730	3.2	29,7–70,8	3–37

**Table 4:** Advantages and disadvantages of fish feed alternatives – oil.

	Advantages	Disadvantages	Ref.
Algae oil	- higher productivity than terrestrial plants - algae can be cultivated in the sea or in wastewater, so there is no need for land and freshwater use - improves the health of aquatic species - improve the appearance of aquatic species which is essential to buyers - rich with omega-3 fatty acids	- high production cost - microalgae have a rigid cell wall which makes digestibility difficult	[20, 27]
Plant oil	- increasing production - high availability - better economic value - rich in omega 6 fatty acids	- poor in omega-3 fatty acids	[20, 49]
Fish oil	- contains polyunsaturated fatty acids, including docosahexaenoic acid (DHA) six times the unsaturated fatty acid	- not as effective as plant oil	[49, 50]

**Algae oils** and fish oils are the primary natural sources of omega-3, but the advantage of algae oils over fish oils is their consistency, sensory properties, and ease of production [47]. The pigment obtained from microalgae improves the color of meat for salmonids and shrimps, increases the antioxidant content in meat, and improves the reproductive health of aquaculture [48].

As **plant oil** most commonly used are soybeans, linseed, rapeseed, sunflower, palm oil, and olive oil in fish feed [49]. As a good alternative, soybeans and rapeseed oil are considered salmon because they are rich in PUFAs (polyunsaturated fatty acids), especially linoleic acid and oleic acid, and do not contain n-3 PUFAs [49]. Replacing 50–60% of fish oil with plant oil results in fish growth processes as with 100% of fish oil [49].

**Fish oil** in traditional aquaculture has been widely used in fish feed, but now the supply of fish oil is dependent on fossil energy and increasing demand for fish [50]. As well as the production of fish oil, contributes to the loss of biodiversity and has an impact on the environment [50].

Table 5 compares the fatty acid values (g/100g) of algae oil, flaxseed oil (vegetable oil) and fish oil.

**Table 5:** Fatty acid composition [51].

	Algae oil	Plant oil – Flaxseed oil	Fish oil
Omega 3 (g/100g)	47.74	37.07	38.65
Omega 6 (g/100g)	7.88	20.26	3.22
Omega-3/Omega-6 ratio	6.06	1.83	12.02
MUFA (g/100g)	3.62	26.37	24.79
PUFA (g/100g)	55.62	57.33	41.78

MUFA - monounsaturated fatty acids.

PUFA - polyunsaturated fatty acid.

The nutritional value and quality of the feed are essential for fish feed and the physical properties of the feed, as physical properties are more important for aquatic animals than for terrestrial animals [19]. When choosing new feed ingredients, it is necessary to look at how this affects the technical properties of fish feed.

## 4 Conclusion

Population growth also leads to an increase in demand for food. This calls for more sustainable feed. In this case study, alternatives used in fish feed production were compared. The composition of fish feed has changed over the years, as in 1990, the main ingredients of fish feed were fishmeal and fish oil. Nowadays, fish feed ingredients have changed, and vegetable proteins and vegetable oils predominate.

Protein from Black Soldier Fly, mealworms, soybean and fishmeal were compared qualitatively and quantitatively as protein sources for fish feed production. One of the most important factors is sustainability. Black Soldier Fly and mealworms are sustainable alternatives as, for example, food leftovers can be used in the cultivation process and effectively converts low-quality organic matter into high-quality proteins and fats. Soybean and Fishmeal are not considered as sustainable alternatives. However, soybean has the benefit of price, which is a disadvantage for the other alternative. Also, Black Soldier Fly and mealworms alternatives is a good source of minerals and vitamins, but needs to be in balance in fish feed because unbalanced diet can cause negative aspect on fish growth.

Comparing the mineral values of protein alternatives, fishmeal is the best performer for zinc (mg/kg), calcium (mg/kg), phosphorus (mg/kg), sodium (mg/kg) and copper (mg/kg). Black Soldier Fly has high values for magnesium (mg/kg) and manganese (mg/kg) and the soybean alternative has better values for iron (mg/kg) and potassium (mg/kg). Minerals affect the health of the fish and subsequently the health of humans. For example, calcium affects bone health in fish and humans, iron affects fish biological reactions and in humans affects production of hemoglobin and potassium affects fish acid-base balance and osmoregulation, but in humans affects the nervous system, muscle function and heart rate.

Algae oil, vegetable oil, and fish oil were compared as oil alternatives for fish feed. The benefits of algae oil are high productivity, improved health and - improve the appearance of aquatic species, as well as a high Omega-3 value. However, the disadvantages are cost and rigid cell wall which makes digestibility difficult. Vegetable oil also increases productivity, is a good alternative from an economic point of view and has a high Omega 6 value. However, it has the disadvantage of being low in Omega 3. Fish oil has the advantage of being rich in fatty acids and the disadvantage of not being as effective as vegetable oil.

Comparing the quantitative data on the fatty acid content of the oil alternatives shows how the qualitative comparison matches the quantitative one. Algae oil has the highest Omega 3 value of the alternatives and vegetable oil, which in this case is flaxseed oil has the lowest Omega 3 value. However, flaxseed oil has the highest Omega 6 value and fish oil the lowest Omega 6 value. Monounsaturated fatty acids (MUFA) are most abundant in flaxseed oil, which is slightly higher than fish oil. Polyunsaturated fatty acids (PUFA) are highest in flaxseed oil and only slightly lower in algae oil. Fatty acids in fish are influenced not only by species and environmental factors, but also by diet. These fatty acids are also important for humans, as fish are the main source of PUFAs.

The composition of fish feed has changed over the years. The nutrients provided by a fish’s diet are strongly influenced by how the fish are fed. Therefore, fish are not only a source of protein but also of vitamins and nutrients, and the enrichment of fish feed needs to be further improved to produce a product with improved properties. In 1990, fishmeal and fish oil dominated, while in 2020, plant resources such as protein and oil dominated fish feed. These changes have come about as fishmeal and fish oil be more sustainable, efficient, and cost-effective alternatives. Alternatives also improve the composition of the feed and the appearance of the final product, which is an essential factor for the consumer.

## Declarations

### Author contribution statement

Beate Zlaugotne: Conceived and designed the experiments; Performed the experiments; Analyzed and interpreted the data; Contributed reagents, materials, analysis tools or data; Wrote the paper.

Jelena Pubule & Dagnija Blumberga: Conceived and designed the experiments; Wrote the paper.

### Funding statement

This work was supported within the framework of the European Regional Development Fund project No. 1.1.1.5/17/I/002 “Integrated national level measures for strengthening interest representations for research and development of Latvia as part of European Research Area” by funding project No. 23-11.17e/21/165 “Non-Food Organic Resources-based feeds optimised for salmon until post-smolt stages”( NON-Fôr).

### Data availability statement

Data included in article/supp. material/referenced in article.

### Declaration of interests statement

The authors declare no conflict of interest.

### Additional information

No additional information is available for this paper.
